# Intracellular Autofluorescence as a New Biomarker for Cancer Stem Cells in Glioblastoma

**DOI:** 10.3390/cancers13040828

**Published:** 2021-02-16

**Authors:** Joana Vieira de Castro, Céline S. Gonçalves, Eduarda P. Martins, Irene Miranda-Lorenzo, Mariana T. Cerqueira, Adhemar Longatto-Filho, Afonso A. Pinto, Rui L. Reis, Nuno Sousa, Christopher Heeschen, Bruno M. Costa

**Affiliations:** 1 Life and Health Sciences Research Institute (ICVS), School of Medicine, Campus Gualtar, University of Minho, 4710-057 Braga, Portugal; joana.castro@i3bs.uminho.pt (J.V.d.C.); celinegoncalves@med.uminho.pt (C.S.G.); id8266@alunos.uminho.pt (E.P.M.); longatto@med.uminho.pt (A.L.-F.); njcsousa@med.uminho.pt (N.S.); 2 ICVS/3B’s-PT Government Associate Laboratory, 4710-057/4805-017 Braga/Guimarães, Portugal; mariana.cerqueira@i3bs.uminho.pt (M.T.C.); rgreis@i3bs.uminho.pt (R.L.R.); 3 Stem Cells and Cancer Group, Molecular Pathology Programme, Spanish National Cancer Research Centre (CNIO), 28029 Madrid, Spain; irenemiranda82@gmail.com (I.M.-L.); christopher.heeschen@sjtu.edu.cn (C.H.); 4 3B’s Research Group, I3Bs—Research Institute on Biomaterials, Biodegradables and Biomimetics, University of Minho, Headquarters of the European Institute of Excellence on Tissue Engineering and Regenerative Medicine AvePark, Zona Industrial da Gandra, 4805-017 Barco, Portugal; 5 Molecular Oncology Research Center, Barretos Cancer Hospital, Barretos 14784-400, Brazil; 6 Medical Laboratory of Medical Investigation (LIM) 14, Department of Pathology, Medical School, University of São Paulo, São Paulo 01246-903, Brazil; 7 Department of Neurosurgery, Hospital de Braga, 4710-243 Braga, Portugal; afonso.pinto@hb.min-saude.pt; 8 Center for Single-Cell Omics & State Key Laboratory of Oncogenes and Related Genes, Shanghai Jiao Tong University School of Medicine, Shanghai 200025, China

**Keywords:** cancer stem cells, glioblastoma stem cells, autofluorescence, biomarker, vitamin B2

## Abstract

**Simple Summary:**

Since glioblastoma stem cells (GSCs) have paramount roles in tumor initiation, progression, recurrence, and therapy resistance, innovative approaches to specifically identify and isolate GSCs in a straightforward manner would be invaluable both for clinical and scientific applications. We demonstrate here that glioblastoma tumors have a subpopulation of cells with intracellular autofluorescence that display all the hallmark features of GSCs, and establish this biomarker as a new, simple, rapid and inexpensive way to identify and isolate this highly aggressive subpopulation of cells. Our findings are a great contribution to the neuro-oncology field, as they allow further studies on the molecular basis of GSCs, which may ultimately contribute to the identification of novel therapeutic targets and the development of smarter treatments to eliminate these cells.

**Abstract:**

The identification of cancer stem cells (CSCs), which are implicated in tumor initiation, progression, therapy resistance, and relapse, is of great biological and clinical relevance. In glioblastoma (GBM), this is still a challenge, as no single marker is able to universally identify populations of GBM cancer stem cells (GSCs). Indeed, there is still controversy on whether biomarker-expressing cells fulfill the functional criteria of bona fide GSCs, despite being widely used. Here, we describe a novel subpopulation of autofluorescent (Fluo^+^) cells in GBM that bear all the functional characteristics of GSCs, including higher capacity to grow as neurospheres, long-term self-renewal ability, increased expression of stem cell markers, and enhanced in vivo tumorigenicity. Mechanistically, the autofluorescent phenotype is largely due to the intracellular accumulation of riboflavin, mediated by the ABC transporter ABCG2. In summary, our work identifies an intrinsic cellular autofluorescent phenotype enriched in GBM cells with functional stem cells features that can be used as a novel, simple and reliable biomarker to target these highly malignant tumors, with implications for GBM biological and clinical research.

## 1. Introduction

Gliomas are the most common primary tumors of the central nervous system, accounting for almost 80% of primary brain malignancies, of which glioblastoma (GBM) is the most aggressive subtype [1,2,3]. Despite several advances in the field of neuro-oncology and the use of a multimodal treatment approach, including surgery, radiotherapy, and chemotherapy, the prognosis of GBM patients has remained very poor, with a median survival of ~15 months [4,5].

Recent evidence suggests that intratumor heterogeneity and poor response to current therapies are at least in part related to the existence of cancer stem cells (CSCs) in GBM [6,7,8,9,10,11,12]. These cells bear important characteristics similar to normal stem cells, most notably their unlimited self-renewal capacity, and have been associated with cancer initiation, progression, resistance to therapy, and relapse [6]. CSCs have been identified and characterized in several cancer types, including glioma. Human brain tumor stem cells (BTSCs) were originally isolated from primary tumors by cell sorting based on their CD133 membrane expression [7,8]. Functionally, CD133^+^–tumor cells were considered to represent CSCs due to their self-renewal ability as evidenced by the formation of anchorage-independent neurospheres, their high proliferation potential, and their differentiation capacity [7]. CD133^+^–BTSCs also displayed enhanced in vivo tumorigenicity in immunocompromised mice, providing strong evidence for the functional distinctness of CSCs in brain tumors [8]. Several subsequent studies corroborated the existence of BTSCs, as well as their increased resistance to radiotherapy and chemotherapy with temozolomide (TMZ) [9,10,11,12].

Due to the pronounced heterogeneity of GBMs, many additional cell surface markers besides CD133 [7,13,14] have been proposed to identify GBM stem cells (GSCs), including CD15 (SSEA–1), CD44, A2B5 epitope, integrin α6, CD90, L1CAM, CXCR4, CD44+ID1 (reviewed in [15]). However, the clinical utility of all these markers can be limited as their expression may only be detectable in a subset of GBMs, is not exclusive of GSCs, and can be altered in response to changes in environmental conditions and sample preparation [16,17,18,19,20]. As such, a universally applicable marker for highly malignant GSCs still remains to be defined. Instead of artifact–prone surface markers, such GSC biomarkers should rather be based on the functional properties of GSCs.

Recently, an intrinsic autofluorescent phenotype has been described for CSCs derived from various human epithelial solid tumors, such as pancreatic ductal adenocarcinoma, colorectal carcinoma, hepatocellular carcinoma and non–small–cell lung carcinoma [21]. While GBMs are phenotypically rather distinct from those carcinomas, herein we demonstrate that intracellular autofluorescence also exists in a subset of GBM cells and is a new biomarker for GSCs, improving their specific identification and isolation, allowing subsequent characterization studies and providing a straightforward method for their tracking during clinical recurrence and treatment follow-up.

## 2. Materials and Methods

### 2.1. Cell Culture

Six different GBM culture models were used: one established human GBM cell line (U251 (*IDH*–wildtype, *MGMT* promoter partially methylated, and *TERT* promoter mutation C228T); obtained from ATCC, Manassas, VA, USA–STR analyses were performed to confirm their authenticity), and 5 human patient-derived primary GBM cultures (GBML1, GBML12, GBML18 (*IDH*–wildtype, *MGMT* promoter unmethylated, and *TERT* promoter mutation C250T), GBML19, and GBML42 (*IDH*–wildtype, *MGMT* promoter partially methylated and *TERT* promoter mutation C228T)), established in our lab as previously described [22].

GBML1, GBML12, GBML19, and GBML42 cultures were maintained in Roswell Park Memorial Institute (RPMI) 1640 (Biochrom, Cambridge, UK), and GBML18 and U251 cultures were maintained in Dulbecco’s Modified Eagle Medium (DMEM; Biochrom). All cultures were supplemented with 10% fetal bovine serum (FBS; Biochrom) and 1% penicillin and streptomycin (Pen/Strep; Gibco, Gaithersburg, MD, USA). Neurospheres were cultured in NeuroCult NS–A Proliferation Kit (Life Technologies, Carlsbad, CA, USA) supplemented with 20 ng/mL epidermal growth factor (EGF; Invitrogen, Carlsbad, CA, USA), 20 ng/mL basic fibroblast growth factor (b–FGF; Invitrogen) and 1% B27 (Invitrogen). In both conditions, adherent and neurospheres cultures were incubated at 37 °C in a humidified atmosphere containing 5% (*v/v*) CO_2_.

### 2.2. Flow Cytometry Analysis

GBM cells were resuspended in FACS flow buffer (BD Biosciences, San Jose, CA, USA) with DAPI (for exclusion of dead cells; 1:1000) before flow cytometry analysis using FACS Canto II (BD Biosciences). In order to identify autofluorescent (Fluo^+^) cells, GBM cells were excited with a 488 nm blue laser and selected as the intersection with filters 530/40 and 580/30 (Figure S1A).

To characterize autofluorescent cells, human primary GBM cultures (GBML1, GBML18, and GBML42) were analyzed by flow cytometry for the expression of CSCs surface markers. Briefly, 1 × 10^5^ cells were incubated with the suitable dilution of appropriate isotype-matched control or specific antibody in 100 µL of PBS for 30 min at 4 °C in the dark. Antibodies used were anti–CD133/1 (1:10; Miltenyi Biotec, Madrid, Spain), anti–CD15 (1:10; BD Biosciences), and anti–CXCR4 (1:10; BD Biosciences). All antibodies were APC–conjugated. Cells were resuspended in 200 µL of FACS flow buffer (BD Biosciences) with DAPI and analyzed by FACS Canto II. Data was analyzed with FlowJo 10.0 software.

### 2.3. Cell Sorting

Before sorting, GBM cultures were incubated overnight with 40 µM of Riboflavin (RBF, Sigma–Aldrich, St. Louis, MO, USA) in a humidified atmosphere at 37 °C and 5% (*v/v*) CO_2_.

Nonautofluorescent cells (Fluo^−^) and autofluorescent cells (Fluo^+^) of human GBM cultures were sorted using a FACS Aria III equipment (BD Biosciences) and correspondent data was analyzed by FACS Diva 7 software (BD Biosciences). Before cell sorting, cell lines were resuspended at a concentration of 5 × 10^6^ cells/mL in sorting buffer (PBS 1×; 3% FBS (*v/v*); 3 mM EDTA (*v/v*)) and filtered through a 40 μM strainer (BD Biosciences) to eliminate cell clumps. Cells were then sorted through a 100 μM nozzle at a sheath pressure of 20 psi. A yield sorting modality (Yield mask sorting for FACS Aria III) was chosen. Gating strategy for sorting was performed as indicated in Figure S1B. To obtain a pure Fluo^−^ subpopulation during sorting procedures an appropriate distance between gates for Fluo^+^ and Fluo^−^ cells is required. Sorted cells (Fluo^−^ and Fluo^+^ subpopulations) were collected in 5 mL polypropylene tubes (BD Biosciences) containing 1 mL collection medium (DMEM or RPMI supplemented with 20% FBS) and transferred to cell culture flasks with prewarmed media (DMEM or RPMI supplemented with 10% FBS and 2% Pen/Strep).

### 2.4. Neurosphere Formation Assay

Neurospheres were generated by culturing 1.5 × 10^3^ human primary GBM cells in Neurospheres media in 24–multiwell plates (0.5 mL/well). Cells were incubated in a humidified atmosphere at 37 °C and 5% (*v/v*) CO_2_. Neurospheres were supplemented with fresh media every 4 days (250 µL/well). After 21 days, the number of neurospheres was counted and pictures were taken. For serial passaging, neurospheres were harvested and dissociated with accutase (EMD Millipore, Burlington, MO, USA) every 21 days. The content of Fluo^+^ cells in neurospheres was evaluated by flow cytometry as described above.

### 2.5. In Vitro Limiting Dilution Assay (LDA)

Multiple serial dilutions from 4 × 10^4^ cells/mL of both Fluo^−^ and Fluo^+^ GBM cells were performed, in 96-well plates. At the end, in each well, cell densities ranged from 1000 to 1 cells in 100 µL of neurospheres media [23]. Cultures were maintained in a humidified atmosphere at 37 °C and 5% (*v/v*) CO_2_, and were supplemented with fresh media every 4 days. After 21 days, the fraction of wells not containing neurospheres was obtained for each condition and plotted against the initially plated cellular density. Stem cell frequencies and statistical significance was calculated using the ELDA software (available at http://bioinf.wehi.edu.au/software/elda/, accessed on 23 August 2019).

### 2.6. Temozolomide (TMZ) and Radiation Treatment

For TMZ (Sigma-Aldrich) treatment, primary GBM cultures (GBML1, GBML18, GBML19 and GBML42) were plated in T25 cm^2^ flasks at an initial density of 1.5 × 10^5^ cells and treated for 9 days with TMZ (600, 500, 850 and 400 μM, respectively) or vehicle (1% DMSO). Culture medium containing TMZ or vehicle was renewed every 3 days. At each timepoint (3, 6 and 9 days), total cells were trypsinized and the percentage of Fluo^+^ cells was evaluated by flow cytometry as described above.

For irradiation treatment, 1.5 × 10^5^ cells of GBML1, GBML18, GBML19 and GBML42 cells were plated in 35 mm diameter plates, and were irradiated with 2, 4, 6, 8 and 10 Gy at 1.94 Gy/min, at room temperature in a 137Cs irradiator (Shepherd Mark-I (model SN1068); J. L. Shepherd and Assoc., San Fernando, CA, USA). Subsequently, cells were washed once with PBS and fresh media was added to the plates that were maintained in a humidified atmosphere at 37 °C and 5% (*v/v*) CO_2_. After three days, total cells were trypsinized and the percentage of Fluo^+^ cells was evaluated by flow cytometry as described above.

### 2.7. RNA Extraction and qRT-PCR

Total RNA from FACS–sorted human primary GBM cultures was extracted with Trizol (Invitrogen) according to the manufacturer’s instructions. cDNA synthesis was performed using 1 µg of total RNA with High Capacity cDNA Reverse Transcription Kit (Applied Biosystems, Foster City, CA, USA). Gene-specific mRNA levels were assessed by quantitative real-time PCR (qRT–PCR) in a real-time thermocycler (CFX96; Bio–Rad, Hercules, CA, USA) using Fast SYBR Green (Qiagen, Hilden, German) according to the manufacturer’s instructions, by the 2^ΔΔCt^ method. The list of primers used can be found in Table S1.

### 2.8. Riboflavin, Fumitremorgin C, and Basal Medium Treatments

For riboflavin (RBF) and fumitremorgin C (FTC, Sigma) treatments, GBM cultures were plated at an initial density of 1 × 10^4^ cells/well, in 6-multiwell plates, in duplicate, and incubated in a humidified atmosphere at 37 °C and 5% (*v/v*) CO_2_. After 24 h, fresh media (control medium basally containing 0.13 µM of RBF) or fresh media supplemented either with RBF (40 µM) or FTC (5 µg/mL) was added to the respective wells.

For basal medium treatment, 4 × 10^4^ GBM cells were plated in 6–well plates, in duplicate, and incubated in a humidified atmosphere at 37 °C and 5% (*v/v*) CO_2_. After 24 h, cells were washed twice with PBS, and fresh media (control condition), basal media (DMEMgfp-2, Evrogen cat. #MC102, Moscow, Russia; medium without vitamins) or basal media containing RBF (40 µM) was added to the respective wells.

In all assays, after 3 days of incubation, GBM cells were trypsinized and washed twice with PBS, and the content of Fluo^+^ cells was evaluated by flow cytometry as described above.

### 2.9. In Vivo GBM Xenografts

For the in vivo LDA subcutaneous assay, five groups receiving 3.0 × 10^5^, 1.0 × 10^5^, 1.5 × 10^4^, 5.0 × 10^3^, or 1.0 × 10^3^ FACS-sorted U251 Fluo^−^ and Fluo^+^ cells were subcutaneously injected in 1:1 (*v/v*) with Matrigel (Corning, Corning, NY, USA) and serum-free DMEM media into the right flank of 12-18-weeks-old NOD.Cg-Prkdc^scid^ Il2rg^tm1Wjl^/SzJ (NSG; The Jackson Laboratory, Bar Harbor, ME, USA) male mice (3–7 mice per group). A positive response was scored when tumor was palpable. Tumor size and body weight were measured at least one time per week. Tumor volume was assessed by measuring the two largest sides and calculated with the formula “*v* = (3.14 × L1 × L1 × L2)/6”. Humane endpoint for sacrifice was applied when one of the mice presented a tumor with ≥2 cm on the larger side. After euthanasia, tumors were collected and weighted.

For the intracranial orthotopic model, a total of 5 × 10^4^ cells (U251 Fluo^−^ or Fluo^+^) were stereotactically injected into the brain striatum (1.8 mm mediolateral, 0.1 mm anteroposterior, and 2.5 mm dorsoventral from the bregma; using a digital 3–axis stereotaxic apparatus; Stoelting, Dublin, Ireland) of 12–weeks–old NSG male mice (6 per group). Mice were anesthetized with a mixture of ketamine (75 mg/kg) and medetomidine (1 mg/kg). Butorphanol (5 mg/kg) was used as analgesia. Cells were resuspended in 5 µL of cold PBS 1× and injected using a 10 µL Hamilton syringe (point style 4 beveled, 26s–gauge needle) at the rate of 1666 µL/min. Animals’ body weight was evaluated ~3 times per week, and general behavior and symptomatology daily. Humane endpoint for sacrifice was established as severe weight loss (>30% of their total body weight relative to the highest body weight value). All brains were collected for histological and molecular analyses. 

All animals were maintained under standard laboratory conditions, including 12 h light/dark artificial cycle, controlled ambient temperature (21 ± 1 °C), and relative humidity of 50–60%. Sentinel mice housed in the same room were used to confirm specific pathogen-free health status, according to FELASA guidelines. During experiments, mice were always manipulated in a flow hood chamber, except during surgery.

### 2.10. Immunohistochemistry

Tissues sections were deparaffinized and rehydrated by xylene and ethanol series. Sodium citrate buffer (10 mM, 0.05% Tween 20, pH 6) was used for antigen retrieval. Endogenous peroxidase activity was blocked with 3% H_2_O_2_ in TBS for 10 min. Ki-67 (#550609, BD Biosciences; 1:200), Nestin (#MAB5326, EMD Millipore; 1:100) and Sox2 (#AB5603, EMD Millipore; 1:500) immunohistochemical staining was performed based on the streptavidin–biotin-–peroxidase complex principle using the LabVision kit (UltraVision Large Volume Detection System Anti-polyvalent, HRP, Fisher Scientific, Hampton, NH, USA) according to the manufacturer’s instructions. Regarding Ki–67 staining, tissues were permeabilized using TBS-Tween 0.5%, for 10 min, before antigen retrieval. For all staining DAB substrate (DAKO, Santa Clara, CA, USA) was used as chromogen, followed by counterstaining with hematoxylin.

### 2.11. Statistical Analyses

All statistical analyses were performed using GraphPad Prism 6.0 (GraphPad software, Inc., San Diego, CA, USA). To assess the statistical differences between groups in the in vitro assays, unpaired Student’s *t*-test analysis was used. Overall survival of orthotopic GBM xenografted mice was compared between groups (Fluo^−^ vs. Fluo^+^) by the log-rank test and plotted as Kaplan–Meier curves. Results are presented as normalized means ± standard deviations (SD), and statistical significance was defined as *p* < 0.05 for a 95% confidence interval.

## 3. Results

### 3.1. Identification of Autofluorescent Cells in Primary GBM Cultures

**Figure 1:**
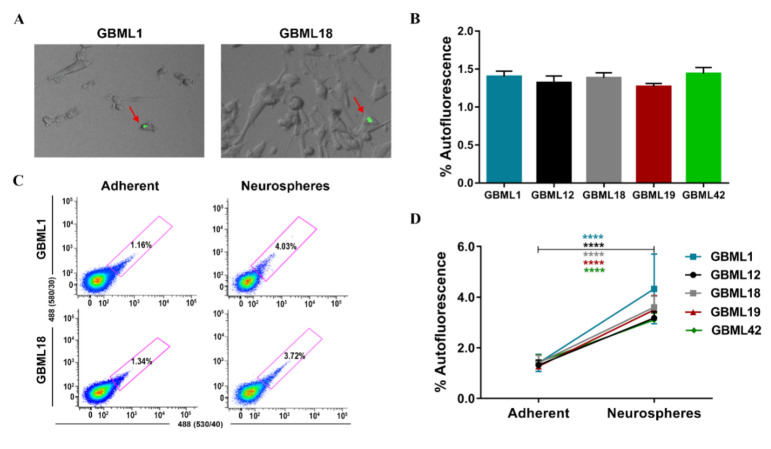
Primary GBM cultures present autofluorescent cells in adherent and neurosphere conditions. (**A**) Representative images of autofluorescent (Fluo^+^) cells (arrows) in human primary GBM cultures, GBML1 and GBML18, grown in adherent conditions. (**B**) Flow cytometry quantification of the percentage of Fluo^+^ cells across five independent human primary GBM cultures propagated in adherent conditions. (**C**) Representative flow cytometry plots indicating the percentage of Fluo^+^ cells in GBML1 and GBML18 human primary cultures, grown in adherent (left) and neurosphere (right) conditions. (**D**) Percentage of autofluorescence across five human primary GBM cultures grown as adherent cells or neurospheres (*n* ≥ 3). Data from (**B**,**D**) panels are represented as the mean ±SD of at least three independent experiments (**** *p* ≤ 0.0001); panels in (**C**) are representative plots of at least three independent experiments.

First, we analyzed the putative presence of autofluorescent (Fluo^+^) cells in two human primary GBM cultures (GBML1 and GBML18) expanded in two–dimensional adherent conditions. Using confocal microscopy, both cultures presented a rare fraction of cells displaying intrinsic green fluorescence (Figure 1A, arrows). Subsequently, a panel of five human primary GBM cultures (GBML1, GBML12, GBML18, GBML19, and GBML42) was investigated for the presence of Fluo^+^ cells using flow cytometry. Consistently, all tested GBM cultures presented a small percentage of Fluo^+^ cells, even when cultured in adherent conditions (Figure 1B). Interestingly, when transferred to tridimensional anchorage–independent and GSC-enriching neurosphere conditions, the percentage of Fluo^+^ cells increased significantly, ranging from 1.27 ± 0.33% in adherent conditions to 3.89 ± 1.11% in neurosphere conditions (Figure 1C,D; *p* ≤ 0.0001 for all tested cultures), suggesting that these autofluorescent cells may have increased stemness properties.

### 3.2. Autofluorescent Cells Present Hallmark Characteristics of GBM Stem Cells

**Figure 2:**
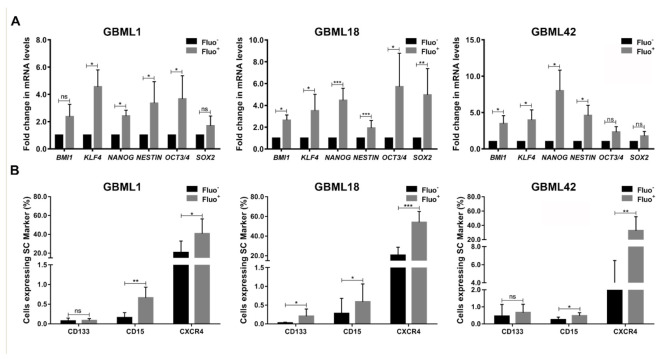
Autofluorescent GBM cells have increased expression of stemness- and pluripotency-associated markers. (**A**) RT–qPCR analyses of pluripotency-associated genes (*BMI1*, *KLF4*, *NANOG*, *NESTIN*, *OCT3/4* and *SOX2*) in fluorescence activated cell sorting (FACS)–sorted Fluo^−^ and Fluo^+^ cells from human primary GBM cultures, GBML1, GBML18 and GBML42. Data shown are normalized for *TBP* expression and represent the fold change between Fluo^+^ vs. Fluo^−^ cells. (**B**) Quantification of flow cytometry analyses for the indicated cell surface stem cell protein markers (CD133, CD15, and CXCR4) in human primary GBM cultures, GBML1, GBML18 and GBML42. Data are represented as the mean ± SD of at least three independent experiments (* *p* ≤ 0.05, ** *p* ≤ 0.01, *** *p* ≤ 0.001).

In order to determine if GBM Fluo^+^ cells display classic features of GSCs, we next analyzed the expression of commonly used pluripotent/stem cell markers. FACS–sorted Fluo^+^ cells from three tested human primary GBM cultures demonstrated significantly increased mRNA expression levels of a variety of pluripotency-associated genes, including *BMI1*, *KLF4*, *NANOG*, *NESTIN*, *OCT3/4,* and *SOX2*, which are frequently reported to be overexpressed in CSCs [24,25,26,27], as compared to their Fluo^−^ counterparts (Figure 2A). Consistently, the expression levels of proteins commonly expressed at the cell surface of GSCs, such as CD133, CD15 and CXCR4 (Figure S2), were also significantly increased in Fluo^+^ cells as compared to Fluo^−^ cells (Figure 2B and Figure S3). Of note, not all tested genes/proteins were consistently upregulated in Fluo^+^ cells of all cultures, which fits well with the widely acknowledged limitation of these biomarkers to be completely specific, if used individually, to identify GSCs.

**Figure 3:**
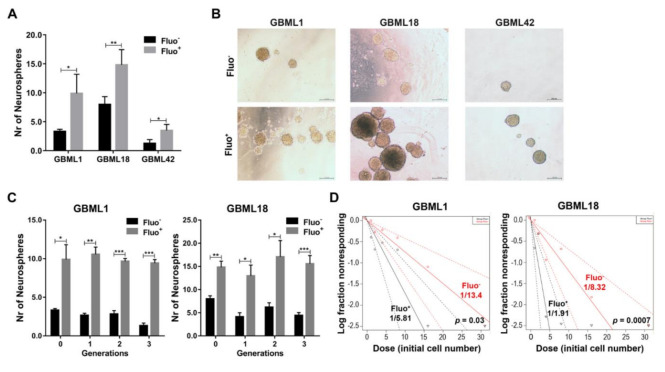
Autofluorescent GBM cells have a higher self–renewal ability. (**A**) Quantification of neurospheres in Fluo^−^ and Fluo^+^ GBM cells sorted from GBML1, GBML18 and GBML42 (*n* ≥ 3; each performed at least in triplicate). (**B**) Representative phase contrast photographs of GBM Fluo^−^ and Fluo^+^ neurospheres from GBML1, GBML18 and GBML42. (**C**) Quantification of neurospheres in Fluo^−^ and Fluo^+^ sorted cells from human primary GBM cultures GBML1 and GBML18, after three consecutive passages/generations (*n* = 3, each performed at least in triplicate). (**D**) Representative LDA analyses of GBML1 and GBML18 Fluo^−^ (red) and Fluo^+^ (black) sorted cells. The graphs are representative of two independent assays for each culture, with similar results. The trend lines represent the estimated active cell frequency (*p* = 0.03 for GBML1 and *p* = 0.0007 for GBML18, likelihood ratio test). Data are representative as the mean ± SD of at least two independent experiments (* *p* ≤ 0.05, ** *p* ≤ 0.01, *** *p* ≤ 0.001).

To complement the biomarker data with functional assays, we sorted Fluo^−^ and Fluo^+^ cells from GBM cultures, and evaluated their capacity to form neurospheres, as clonogenic growth in neurospheres serves as an in vitro marker for self-renewal ability and has been linked to GBM stemness [7,28]. In all tested primary GBM cells, Fluo^+^ cells showed significantly higher capacity to form neurospheres, as compared to their Fluo^−^ counterparts (Figure 3A,B). Moreover, to more stringently assess self-renewal capacity, we also evaluated neurosphere formation over three consecutive passages in sorted GBML1 and GBML18 cells, in which Fluo^+^ cells formed significantly higher numbers and larger-sized neurospheres in serial passages, as compared to Fluo^−^ cells (Figure 3C). Subsequently, an LDA was performed to more comprehensively quantify the neurosphere formation capacity of FACS-sorted Fluo^−^ and Fluo^+^ cells. Concordantly, Fluo^+^ cells displayed a significantly higher frequency of neurosphere formation as compared to Fluo^−^ counterparts (stem cell frequency: GBML1, 1/5.81 for Fluo^+^ versus 1/13.4 for Fluo^−^, *p* = 0.03; GBML18, 1/1.91 for Fluo^+^ versus 1/8.32 for Fluo^−^, *p* = 0.0007; Figure 3D), further highlighting the functional link between the autofluorescence phenotype and GBM cell stemness features.

**Figure 4:**
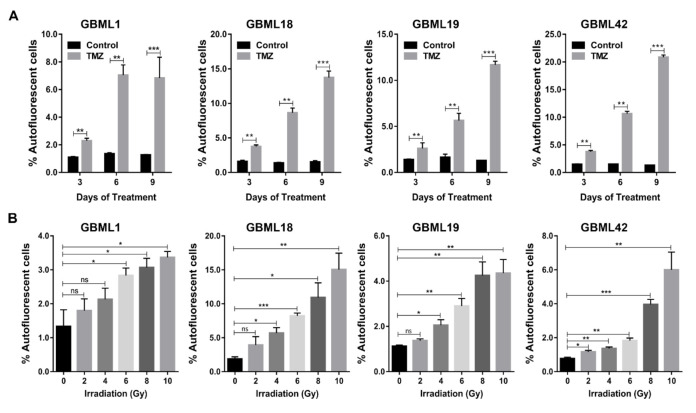
Temozolomide and radiation treatments increase the percentage of GBM autofluorescent cells. (**A,B**) Quantification of Fluo^+^ percentage in control *vs.* Temozolomide (TMZ)–treated primary GBM cultures (GBML1, GBML18, GBML19, and GBML42; (**A**) and in control *vs.* irradiated cells (2, 4, 6, 8, and 10 Gy; (**B**). Data are represented as the mean ± SD of three independent experiments (* *p* ≤ 0.05, ** *p* ≤ 0.01, *** *p* ≤ 0.001).

It has also been widely reported that GSCs are particularly resistant to chemo– and radio–therapy [9,10,11,29,30]. Thus, we treated human primary GBM cultures with TMZ (600, 500, 850, and 400 µM, respectively), the standard chemotherapeutic agents used to treat GBM patients, and evaluated its effect on the population of Fluo^+^ cells. Interestingly, TMZ treatment significantly increased, in a consistent and time–dependent manner, the percentage of Fluo^+^ cells in all primary GBM cultures (Figure 4A and Figure S4A). Similarly, exposure of these human primary GBM cultures to various doses of radiation treatment (0, 2, 4, 6, 8, and 10 Gy) also significantly enriched, in a dose-dependent manner, the subpopulation of GBM Fluo^+^ cells (Figure 4B and Figure S4B). 

Together, our data demonstrate that Fluo^+^ cells in GBM carry essential molecular and functional hallmarks of CSCs, including increased expression of pluripotency-associated genes and stem cell protein markers, enriched capacity to grow as neurospheres, higher self–renewal ability, and are enriched upon treatment with either chemotherapy or radiation.

### 3.3. Riboflavin is a Major Source of Intracellular Autofluorescence in GSCs

**Figure 5:**
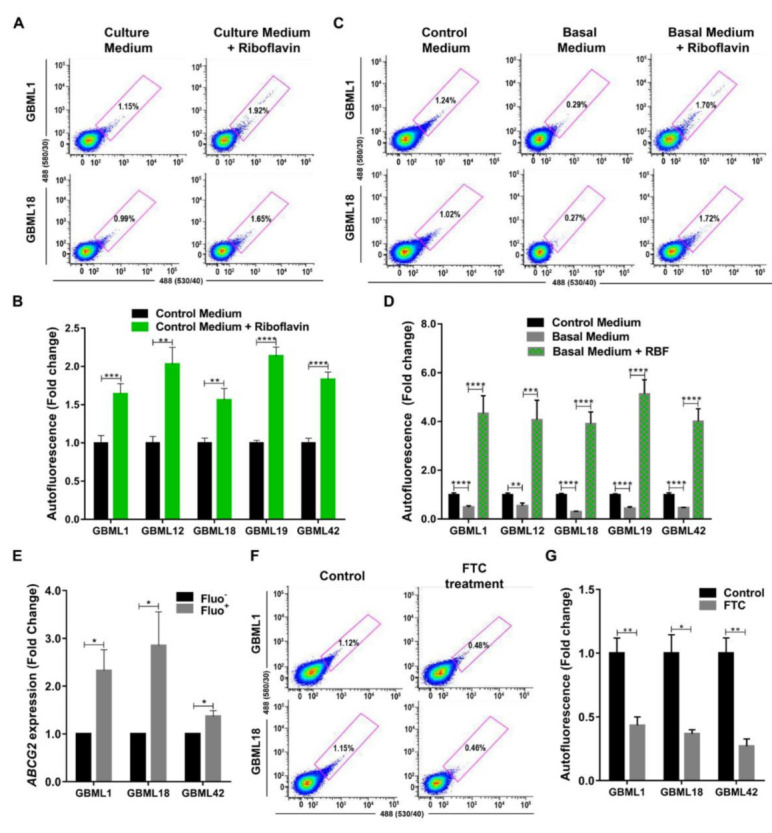
Riboflavin contributes to the autofluorescence phenotype of GBM stem cells. (**A,B**) Representative flow cytometry plots (**A**) and quantification (**B**) of Fluo^+^ cells from primary GBML1 and GBML18 cultures in control medium, or control medium supplemented with 40 μM of riboflavin. (**C,D**) Representative flow cytometry plots (**C**) and quantification (**D**) of Fluo^+^ cells from primary GBML1 and GBML18 cultures in control medium, basal medium (without vitamins), or basal medium supplemented with 40 μM of riboflavin (RBF). (**E**) RT–qPCR analysis of *ABCG2* transporter in Fluo^−^ and Fluo^+^ sorted cells from GBML1, GBML18, and GBML42 primary cultures. Data are normalized for *TBP* expression and represent the relative ratios between Fluo^+^ and Fluo^−^. (**F,G**) Representative flow cytometry analysis (**F**) and quantification (**G**) of autofluorescence in GBML1 and GBML18 cultures treated with 5 µg/mL of fumitremorgin C (FTC). Data from (**B**,**D**,**E**,**G**) panels are represented as the mean ± SD of at least three independent experiments; (**A**,**C**,**F**) panels are representative plots of at least three independent experiments (* *p* ≤ 0.05, ** *p* ≤ 0.01, *** *p* ≤ 0.001, **** *p* ≤ 0.0001).

Previously, the fluorescent vitamin riboflavin (vitamin B2), a substrate for the ABCG2 transporter, has been linked to the autofluorescent phenotype in CSCs from various carcinomas [21]. Therefore, we tested in our five human patient-derived primary GBM cultures and in an established GBM cell line (U251) whether riboflavin levels contribute to the autofluorescent phenotype in GBM. Indeed, treatment with riboflavin led to a significantly increased autofluorescence in all GBM cultures (Figure 5A,B and Figure S5A). To further test this hypothesis, GBM cells were cultured in basal medium (without vitamins) for a short period of 72 h, which was sufficient to consistently detect significant decreases in the percentage of Fluo^+^ cells (Figure 5C,D, and Figure S5B). Concordantly, this effect was completely reversed by the addition of riboflavin to the basal medium (Figure 5C,D and Figure S5B). Together, these data identify riboflavin as a critical contributor to the autofluorescence phenotype in GSCs.

To understand if ABCG2 could be involved in the intracellular transport/accumulation of riboflavin in GSCs, we evaluated putative changes in the expression levels of *ABCG2* mRNA in Fluo^−^ and Fluo^+^ primary GBM cultures. Indeed, *ABCG2* was significantly and consistently overexpressed in the Fluo^+^ subpopulations as compared to their respective Fluo^−^-counterparts (Figure 5E). Concordantly, the pharmacological inhibition of the ABCG2 transporter with FTC, a recognized inhibitor of the ABCG2 transporting activity by allosteric binding to the protein, causing a conformational change that results in the impairment of ABCG2-mediated transport, significantly decreased the percentage of Fluo^+^ cells in all tested primary GBM cultures (Figure 5F,G). Together, these data demonstrate that autofluorescence in GBM occurs in GSCs largely by the ABCG2-mediated transport and intracellular accumulation of riboflavin.

### 3.4. Autofluorescence GSCs are Associated with Tumor Aggressiveness in GBM Xenografts Models

A critical functional hallmark of CSCs is their increased tumorigenicity and association with more aggressive tumors in vivo. To evaluate if the subpopulation of Fluo^+^ cells presents a higher tumorigenic capacity, an in vivo limiting dilution assay was performed by subcutaneously transplanting into NSG mice a decreasing number of FACS-sorted U251 Fluo^−^ and Fluo^+^ cells (3 × 10^5^, 1 × 10^5^, 1.5 × 10^4^, 5 × 10^3^, and 1 × 10^3^). Interestingly, mice injected with Fluo^+^ cells developed tumors more rapidly than mice injected with Fluo^−^ cells (100% and 33% tumor penetrance at day 23, respectively), demonstrating different kinetics of tumor growth (Figure S6A), as reflected in higher tumor-forming frequencies at day 23 of Fluo^+^ cells than their negative counterparts (1/62 253 for Fluo^+^ versus 1/358 220 for Fluo^−^, *p* = 0.0017; Figure S6B). At the humane endpoint of the experiment (day 69), all mice had developed tumors as expected (Figure S6A), but tumors derived from Fluo^+^ cells were significantly larger than those from Fluo^−^ cells (Figure S6C–E).

**Figure 6:**
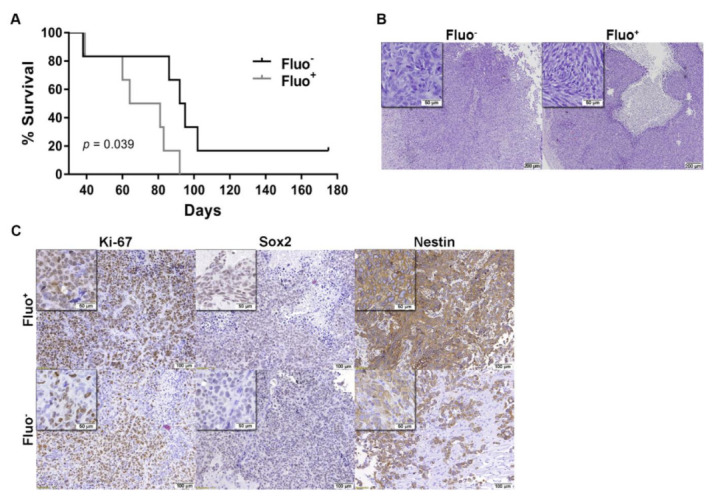
Autofluorescent cells are associated with increased aggressiveness in in vivo GBM xenografts. (**A**) Kaplan–Meier survival curves of NSG mice intracranially injected with Fluo^−^ or Fluo^+^ U251 GBM cells (*n* = 6/group; Log–rank test, *p* = 0.039). (**B**) Representative images of hematoxylin and eosin staining in tissue sections at endpoint. (**C**) Representative images of Ki–67, Sox2 and Nestin.

To further validate that Fluo^+^ cells promote a more aggressive in vivo tumor phenotype, a more relevant intracranial orthotopic human GBM model was tested [12,31,32]. FACS–sorted U251 Fluo^+^ and Fluo^−^ cells were orthotopically injected into the brain striatum of NSG mice, and closely followed for overall survival, the most relevant outcome in the context of highly aggressive GBM. Interestingly, mice bearing tumors originated from Fluo^+^ cells showed a significantly shorter overall survival (median 70 days) as compared to mice injected with Fluo^−^ cells (median 99 days; Log rank test, *p* = 0.039; Figure 6A). Further, hematoxylin/eosin (H&E) analyses confirmed tumor formation and characteristic hallmarks of GBMs, such as pleomorphic and spindle shape tumor cells, high mitotic activity, and prominent nuclear polymorphism in all animals (Figure 6B). Interestingly, compared to Fluo^−^ tumors, tumors derived from Fluo^+^ cells showed increased proliferation indexes as evidenced by Ki–67 staining, as well as the increased expression of the stemness markers as Nestin and Sox2 (Figure 6C). Globally, these data highlight the increased tumor aggressiveness of Fluo^+^ GBM cells in vivo, a hallmark feature of CSCs, being associated with poorer prognosis.

## 4. Discussion

Using a diverse panel of primary human GBM cultures, we identified an intrinsic autofluorescent phenotype in GBM cells, which had the hallmark features of CSCs. We also demonstrated that the underlying mechanism of the increased autofluorescent phenotype was largely due to the accumulation of riboflavin in GSCs. These Fluo^+^ cells could be identified by flow cytometry and isolated by FACS both from primary and established human GBM cell lines, either in adherent monolayers or in tridimensional neurosphere conditions. Importantly, these Fluo^+^ cells are also present in freshly dissociated GBM tumors from patients, as well as in ex vivo xenograft tumors derived from the subcutaneous injection of U251 GBM cells (Figure S7). Additionally, it would be interesting to understand how GBMs from different brain regions may display differential levels of autofluorescence, particularly testing whether tumors resected from so–called stem cell niches are intrinsically enriched for autofluorescence. 

Previously, an autofluorescent phenotype was reported for GBM cells, but it was later demonstrated to be related to the contamination with HEK–293T cells stably expressing GFP [33]. While such scenario would be highly unlikely in the context of the various independent cultures used in our study, and particularly using short-term patient-derived primary cultures, in order to fully exclude such artifacts, STR analyses were performed on all unsorted and sorted Fluo^+^ cells from GBML1, GBML18, GBML42, and U251 GBM cultures, demonstrating that autofluorescent cells are genotypically identical to unsorted bulk cells, and do not present the typical HEK-293T STR marker profile.

This new autofluorescence GSC marker has critical advantages over other currently used biomarkers, such as CD133 or CD15 expression, in that it does not require extensive processing for antibody staining, eliminating any problem associated with epitope recognition. Moreover, autofluorescence allows for the constant monitoring of the GSC state without repetitive staining, thus enabling this technology to be conceptually used for the monitoring of interventions, e.g., intraoperative mapping, drug treatment or irradiation.

GSCs are defined by particular characteristics, including self–renewal capacity, unlimited proliferation, stem cell marker expression, and ability to differentiate into multiple cell lineages. As such, GSCs play a decisive role during tumor initiation and progression [34]. Importantly, our data validate that Fluo^+^ cells possess all of these functional characteristics, including the overexpression of stem and pluripotent-associated markers (Figure 2 and Figure S3); increased self-renewal capacity (Figure 3); and enhanced in vivo tumorigenicity and shorter in vivo survival (Figure 6 and Figure S6). Consistently, we demonstrate that treatment with TMZ or radiation results in the relative enrichment of Fluo^+^ cells in vitro (Figure 4 and Figure S4). Together, our data are in line with the notion that CSCs are resistant to current treatments and subsequently enriched, rendering the tumor more aggressive and resulting in rapid tumor relapse [35,36]. In the future, it will be interesting to develop further in vivo studies to evaluate how Fluo^−^- and Fluo^+^-derived tumors may respond differently to various treatments (e.g., TMZ and/or radiation).

Mechanistically, we demonstrate that the accumulation of riboflavin inside GBM cells significantly contributes to the autofluorescent phenotype (Figure 5A–D and Figure S5), consistent with what has previously been reported for carcinomas [21]. Moreover, we verify that the Fluo^+^ subpopulation overexpresses the *ABCG2* transporter (Figure 5E), a finding in line with previous studies showing that ABCG2 is highly expressed in stem cells, including GSCs [37,38,39]. ABCG2 transporters are not only responsible for secreting riboflavin into the milk in lactating mammary glands, but also for transporting and mediating a marked intravesicular accumulation of riboflavin in ABCG2–overexpressing breast and lung cancer cells [40,41]. This is in agreement with our findings showing that ABCG2 mediates the transport of riboflavin in GSCs. Specifically, we show that pharmacological inhibition of this transporter with FTC leads to a significant decrease in the percentage of Fluo^+^ cells (Figure 5F,G), demonstrating that the autofluorescent phenotype is, at least in part, due to the transport of riboflavin by ABCG2. Notably, in our studies in GBM, this decrease was not complete, raising the possibility that riboflavin may also be transported into the cells by transporters other than ABCG2. In fact, Fu and colleagues showed that riboflavin transporter 2 (RFT2), a human riboflavin transporter, is overexpressed in glioma samples as compared to in the normal brain, and was associated with WHO glioma grade [42]. In addition, the silencing of *RFT2* was associated with inhibition of glioma cell proliferation by promoting apoptosis and cell cycle arrest, a reduced invasion and migration, and a decreased tumor growth in vivo [42]. This same transporter was also described to be overexpressed in esophageal squamous cell carcinoma and involved in regulating cell cycle progression, cell proliferation, energy metabolism, tumorigenicity in vivo, and maintaining normal intracellular flavin status [43]. In the future, it will be important to better clarify the role of riboflavin in the biology of GSCs. In fact, it is known that riboflavin is involved in numerous enzymatic reactions in all forms of life, and performs key metabolic functions by mediating the transfer of electrons in biological oxidation-reduction reactions in bioenergetic metabolism [44,45]. Additionally, it will also be critical to further characterize Fluo^+^ and Fluo^−^ GBM cells by evaluating the signaling pathways and genes that are differentially expressed in these functionally very distinct subpopulations. This could pave the way for identifying novel therapeutic targets and developing more effective treatments to eliminate GSCs. Such therapies targeting Fluo^+^ (GSC) cells could be a promising and rational approach for these very aggressive tumors. For example, GBM patients may also potentially benefit from therapies targeting ABCG2; indeed, some anti-ABCG2 therapies have been recently tested in preclinical and clinical contexts [46,47,48,49,50,51,52,53] and could prove more efficacious in targeting Fluo^+^ GSCs. In addition, the levels of autofluorescence, reflecting the GSC content, may be useful for the follow-up evaluation of tumor response to therapy. Finally, and considering the widely known heterogeneity of GBMs, a deeper molecular analysis of all our primary cultures should be performed.

## 5. Conclusions

In conclusion, our study demonstrates that intracellular autofluorescence can be used as a reliable biomarker to identify, track, and isolate GBM cells with GSC features, impacting on biological research and clinical monitoring and interventions.

## Data Availability

The data presented in this study are available upon request from the authors.

## Supplementary Materials

The following are available online at https://www.mdpi.com/2072-6694/13/4/828/s1, Figure S1: Identification of GBM autofluorescent cells by flow cytometry; Figure S2: Expression of stem cell surface markers (CD133, CD15 and CXCR4) in GBML1 (A), GBML18 (B), and GBML42 (C) cultures; Figure S3: Autofluorescent GBM cells have increased expression of stem cell surface markers; Figure S4: Autofluorescent populations are enriched after chemo- or radiotherapy treatment; Figure S5: Riboflavin is the source of autofluorescent cells in established U251 GBM cell line; Figure S6: Autofluorescent cells are associated with increased tumor growth in vivo; Figure S7: Autofluorescent (Fluo^+^) cells are present in GBM tumors; Table S1: Sequence of primers used for RT–qPCR analyses.
