# Supplementary material for: Intracellular Autofluorescence as a New Biomarker for Cancer Stem Cells in Glioblastoma
Source: Cancers (Basel). 2021 Feb 16;13(4):828. doi: 10.3390/cancers13040828 (PMC7920313; doi:10.3390/cancers13040828)
Supplement: Supplementary file 1 [file cancers-13-00828-s001.pdf]

Supplementary Materials

Intracellular Autofluorescence as a New Biomarker for Cancer Stem Cells in Glioblastoma

Joana Vieira de Castro, Céline S. Gonçalves, Eduarda P. Martins, Irene Miranda-Lorenzo, Mariana T. Cerqueira, Adhemar Longatto-Filho, Afonso A. Pinto, Rui L. Reis, Nuno Sousa, Christopher Heeschen and Bruno M. Costa

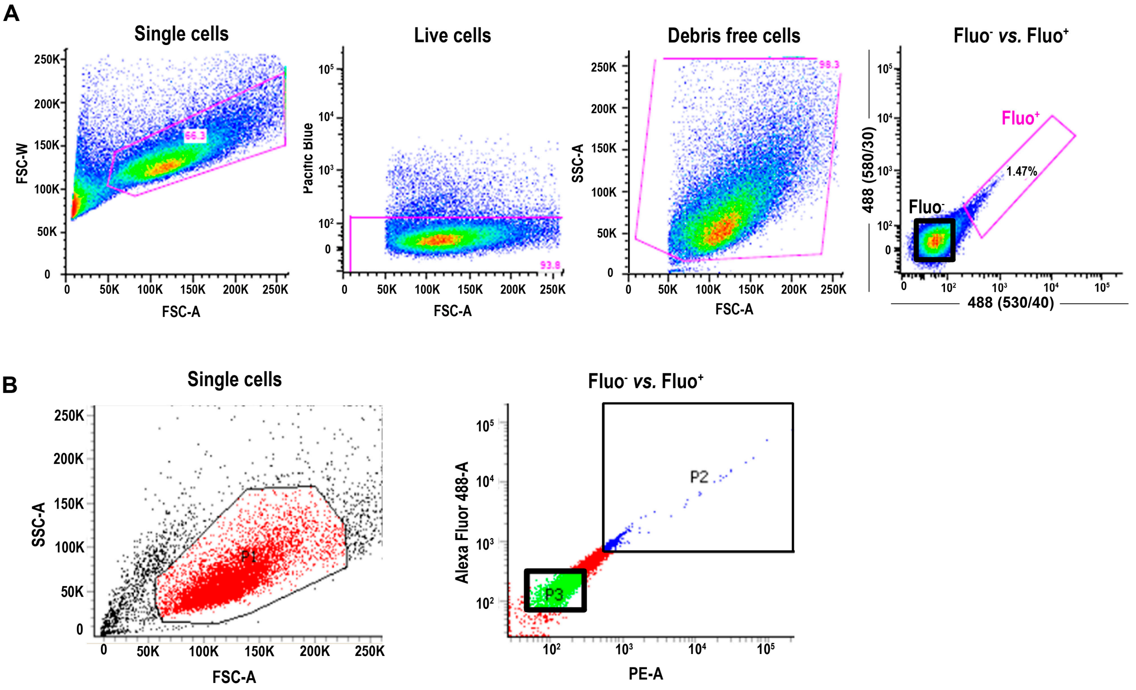

Figure S1: Identification of GBM autofluorescent cells by flow cytometry. (**A**) Representative flow cytometry plots demonstrating the strategy used for the identification of Fluo⁺ cells. These cells are excited with a 488–nm blue laser and selected with the intersection of 530/40 and 580/30 filters, where Fluo⁺ corresponds to autofluorescent subpopulation and Fluo⁻ corresponds to non–autofluorescent cells. (**B**) Gating strategy used for sorting Fluo⁺ and Fluo⁻ cells. A FITC *vs.* PE dot plot was performed, P2 corresponds to Fluo⁺ fraction and P3 corresponds to Fluo⁻ subpopulation.

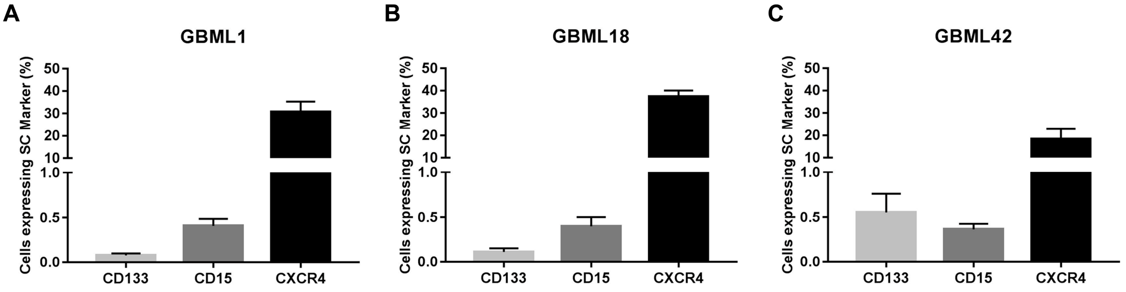

Figure S2: Expression of stem cell surface markers (CD133, CD15 and CXCR4) in GBML1 (**A**), GBML18 (**B**), and GBML42 (**C**) cultures.

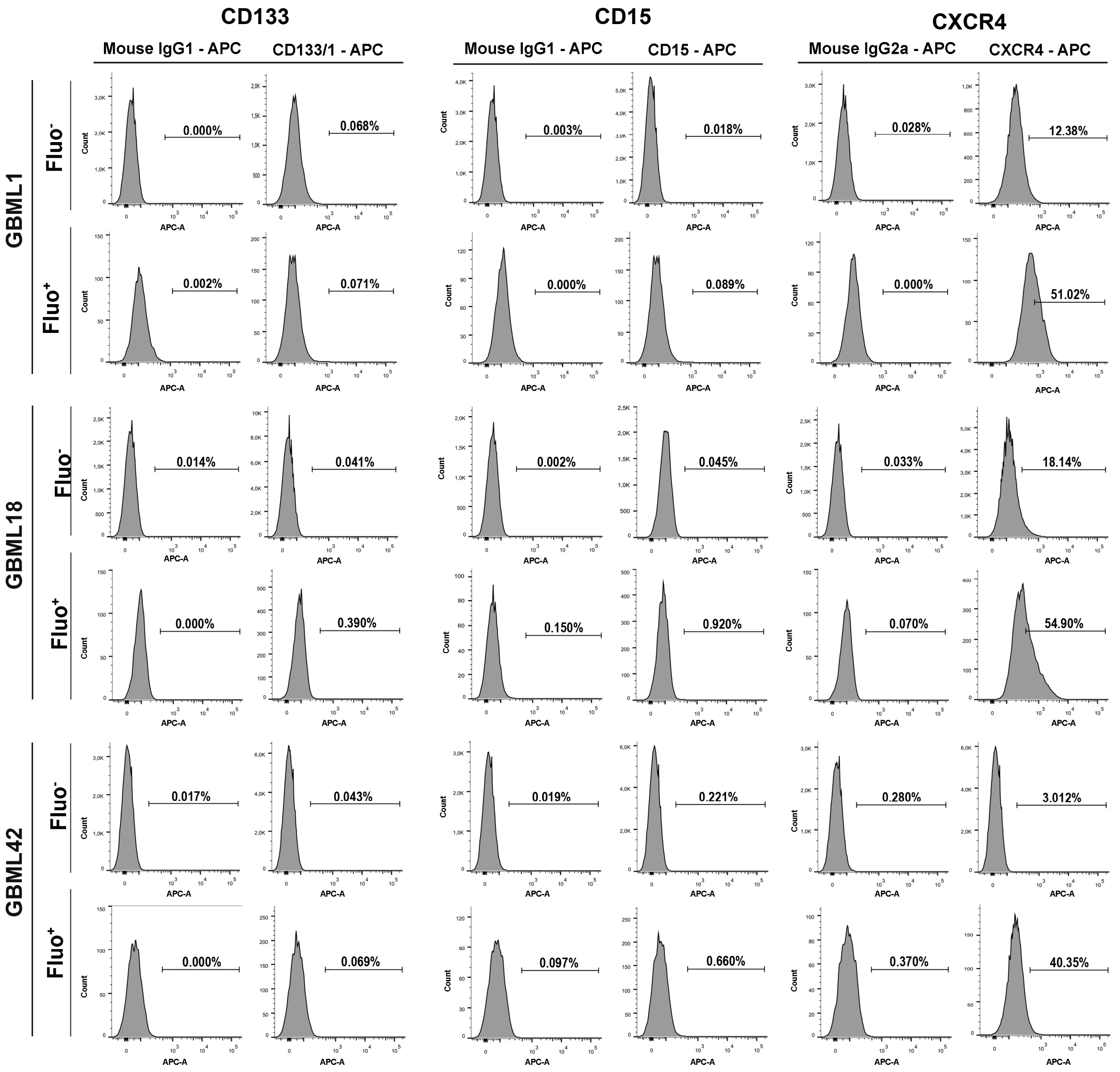

Figure S3: Autofluorescent GBM cells have increased expression of stem cell surface markers. Representative flow cytometry analysis for the indicated stem cell surface markers in Fluo⁺ and Fluo⁻ cells from GBML1, GBML18, and GBML42 cultures.

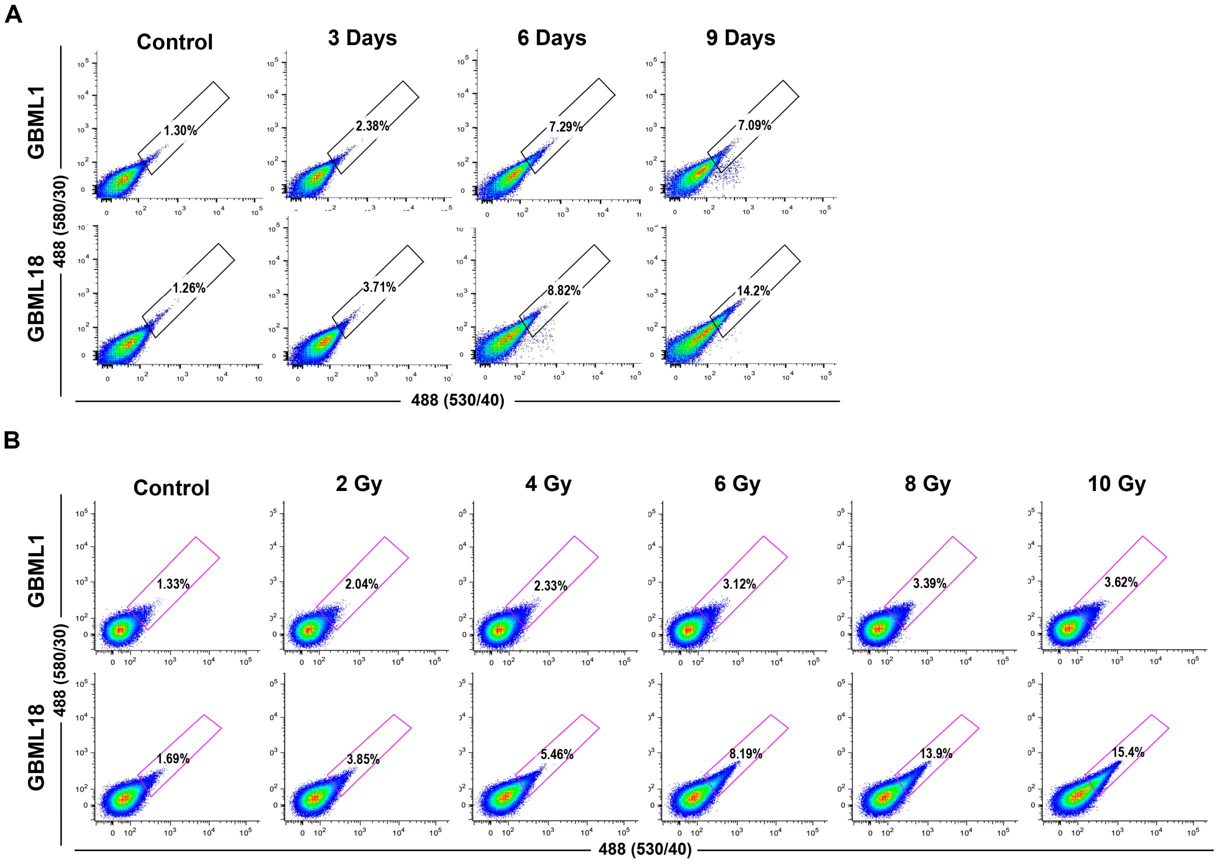

Figure S4: Autofluorescent populations are enriched after chemo– or radio–therapy treatment. (**A,B**) Representative flow cytometry images of autofluorescent cells in human primary GBM cultures (GBML1 and GBML18) in control (DMSO) *vs.* TMZ–treated cells over time (**A**), and in control *vs.* irradiated cells (2, 4, 6, 8, and 10 Gy; **B**).

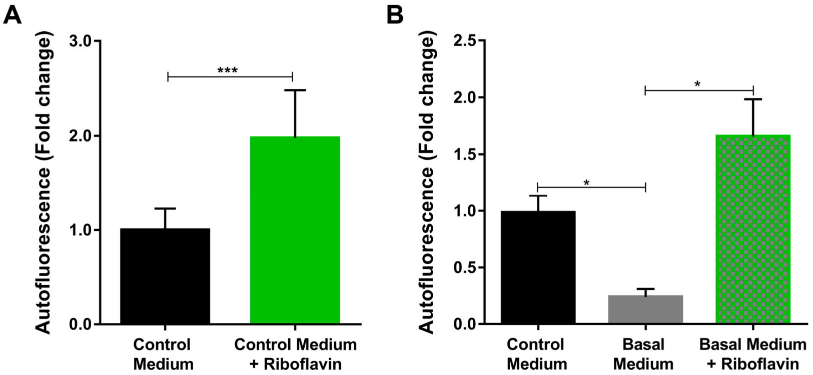

Figure S5: Riboflavin is the source of autofluorescent cells in established U251 GBM cell line. (**A**) Quantification of autofluorescent cells in U251 GBM cell line cultured in control media or control media containing 40 µM of Riboflavin (RBF). (**B**) Quantification of autofluorescent content in U251 GBM cell line cultured in control medium, basal medium (without vitamins), or basal medium supplemented with 40 µM of RBF. Data is represented as the mean ± SD of three independent experiments (*$p \leq 0.05$, ***$p \leq 0.001$).

A

% of tumor formation				
Days after cells injection	23		69	
Grafted cells	Fluo⁻	Fluo⁺	Fluo⁻	Fluo⁺
3.0x10⁵	33.3(1/3)	100.0 (3/3)	100.0 (3/3)	100.0 (3/3)
1.0x10⁵	50.0 (2/4)	75.0 (3/4)	100.0 (4/4)	100.0 (4/4)
1.5x10⁴	0.0 (0/7)	14.3 (1/7)	100.0 (7/7)	100.0 (7/7)
5.0x10³	0.0 (0/7)	0.0 (0/7)	100.0 (7/7)	100.0 (7/7)
1.0x10³	0.0 (0/7)	0.0 (0/7)	100.0 (7/7)	100.0 (7/7)

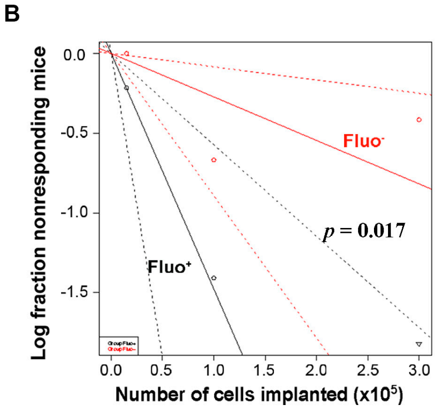

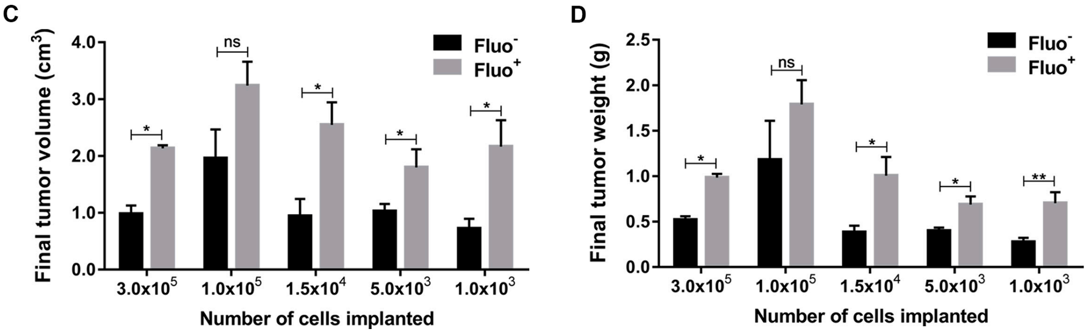

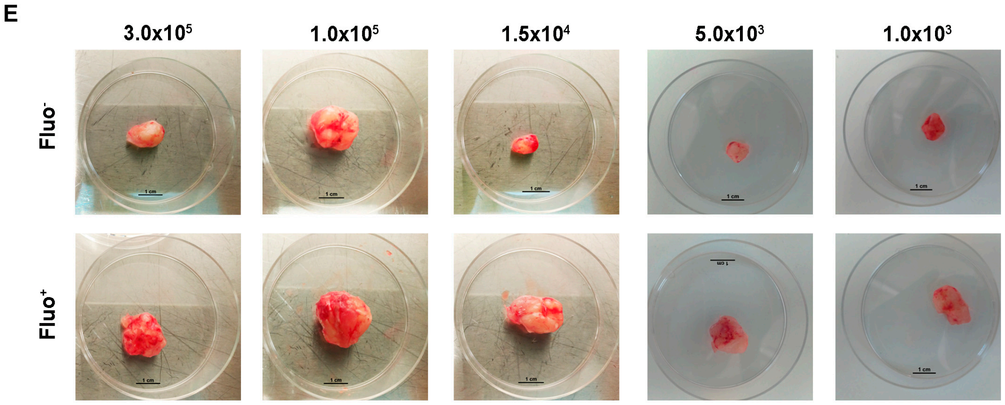

Figure S6: Autofluorescent cells are associated with increased tumor growth *in vivo*. (**A**,**B**) *In vivo* limiting dilution analysis of FACS–sorted Fluo⁻ and Fluo⁺ U251 cells subcutaneously injected at different numbers ($3x10^5$, $1x10^5$, $1.5x10^4$, $5x10^3$, and $1x10^3$) into NSG mice, assessed at days 23 and 69 (endpoint) after tumor implantation. (**A**) Percentage of tumor formation at days 23 and 69. (**B**) Tumor–formation frequency at day 23 was calculated using ELDA software for Fluo⁻ (1/358220) and Fluo⁺ (1/62253; $p = 0.017$, likelihood ratio test). (**C**) Final tumor volumes *in vivo*. (**D**) Final tumor weights *ex vivo*. (**E**) Representative photographs of *ex vivo* tumors derived from U251 Fluo⁻ (**E**, top) and Fluo⁺ (**E**, bottom) GBM cells. Data is represented as the mean ± SD of the mice in each group ($*p \leq 0.05$, $**p \leq 0.01$).

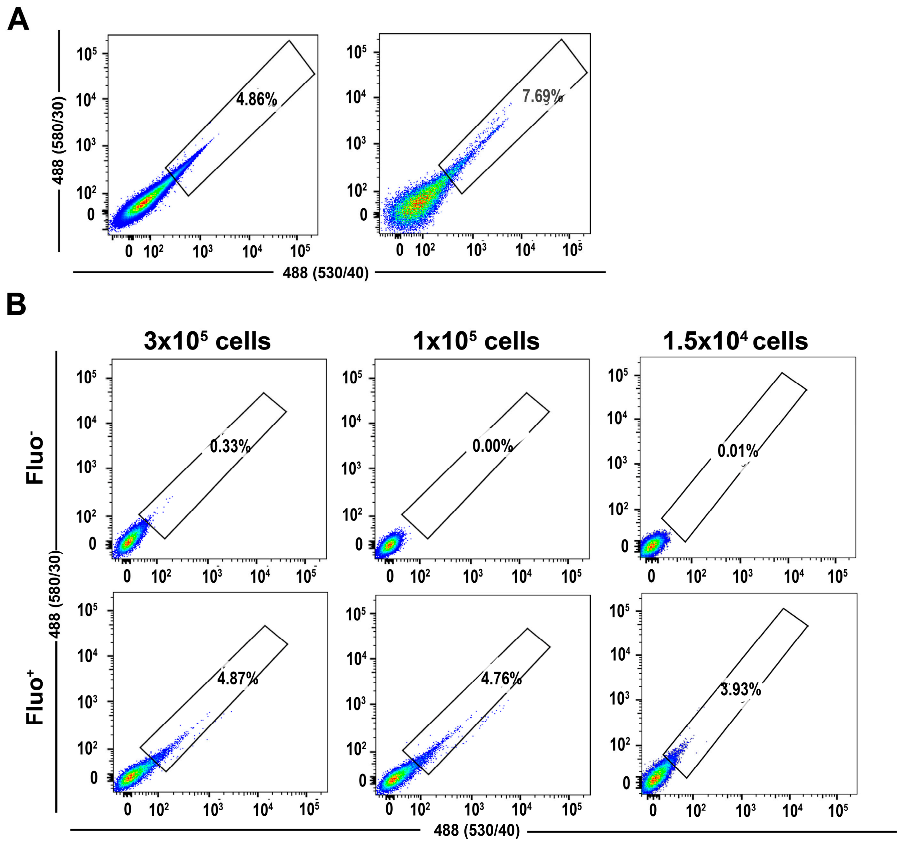

Figure S7: Autofluorescent (Fluo+) cells are present in GBM tumors. Representative flow cytometry data of autofluorescent cells in primary tumors from 2 GBM patients (**A**), as well as from xenograft tumors derived from the subcutaneous injection of FACS–sorted U251 Fluo- and Fluo+ cells (**B**).

Table S1: Sequence of primers used for RT–qPCR analyses.

Gene	Primer Sense	Primer Antisense
ABCG2	TCATGTTAGGATTGAAGCCAAAGGC	TGTGAGATTGACCAACAGACCTGA
BMI1	TTCTTTGACCAGAACAGATTGG	GCATCACAGTCATTGCTGCT
KLF4	ACCCACACAGGTGAGAAACC	ATGTGTAAGGCGAGGTGGTC
NANOG	TGAACCTCAGCTACAAACAGGTG	AACTGCATGCAGGACTGCAGAG
NESTIN	CAGGAGAAACAGGGCCTACA	TGGGAGCAAAGATCCAAGAC
OCT3/4	CTTGCTGCAGAAGTGGGTGGAGGAA	CTGCAGTGTGGGTTTCGGGCA
SOX2	AGAACCCCAAGATGCACAAC	CGGGGCCGGTATTTATAATC
TBP	GAGCTGTGATGTGAAGTTTCC	TCTGGGTTTGATCATTCTGTAG

For all genes, qPCR parameters were as follows: 4 minutes at 94 °C, 40 cycles of denaturation for 30 seconds at 94 °C, annealing for 30 seconds at 60 °C, and extension at 72 °C for 30 seconds, and final extension increasing the temperature in 1 °C each 5 seconds from 65 °C to 95 °C.
